# Spontaneous splenic rupture in a patient with paratyphoid a fever: a case report and literature review

**DOI:** 10.3389/fmed.2026.1882061

**Published:** 2026-07-03

**Authors:** Fangwan Yang, Zhi Kou, Yin Zhang, Xiaohuan Wu, Yuyu Zhu, Man Chen, Hongmei Li, Shenghui Zhang, Shide Lin, Ying Li

**Affiliations:** 1 Department of Infectious Diseases, The Second Affiliated Hospital of Zunyi Medical University, Zunyi, Guizhou, China; 2 Department of Emergency, The Second Affiliated Hospital of Zunyi Medical University, Zunyi, Guizhou, China

**Keywords:** case report, enteric fever complications, *Salmonella paratyphi* A, splenic hemorrhage, spontaneous splenic rupture

## Abstract

**Background:**

Enteric fever, including both typhoid and paratyphoid fevers, is associated with a significant burden of morbidity and mortality in high-epidemic countries. However, as its incidence has declined in most countries, early diagnosis of its complications has become a clinical challenge. Spontaneous splenic rupture (SSR) is a rare but dangerous complication of typhoid fever, and its occurrence in patients with paratyphoid fever remains poorly understood. We report a case of SSR associated with paratyphoid A fever and briefly review the literature.

**Case presentation:**

A 15-year-old male presented with a one-week history of high fever and fatigue complicated by sudden onset of left upper quadrant abdominal pain for 2 days. Physical examination revealed diffuse abdominal tenderness without rigidity; laboratory studies showed leukocytosis and markedly elevated inflammatory markers. Blood cultures revealed *Salmonella enterica serovar* Paratyphi A. Contrast-enhanced computed tomography showed splenic rupture and a subcapsular hematoma, peritoneal and pelvic effusion, and peritonitis. He was hemodynamically stable, so we adopted nonoperative management. The patient recovered uneventfully. A search of English-language literature revealed only four reported SSR cases in typhoid fever and two in paratyphoid fever (one Paratyphi A and one Paratyphi B), with a mortality of 33.3% (2/6). All patients either lived in or had traveled back from high-endemic countries.

**Conclusion:**

SSR is a rare but life-threatening complication of both typhoid fever and paratyphoid fever. Clinical vigilance for this complication should be maintained in patients with suspected enteric fever, even in low-endemic settings.

## Background

Enteric fever is a systemic illness caused by *Salmonella enterica serovars* Typhi (*S.* Typhi) and *S.* Paratyphi. It remains a major health problem worldwide, especially in low- and middle-income countries (1). In 2021, it was estimated that there were 9.3 million cases of enteric fever and 107,500 deaths globally (2). The prevalence of enteric fever varies greatly among countries, with high endemicity in South Asia, Southeast Asia, and sub-Saharan Africa and low prevalence in developed countries (3). In China, the prevalence of typhoid/paratyphoid fever has consistently declined from 0.844 to 0.443 per 100,000 population from 2011 to 2022 (4).

The clinical course of enteric fever can be complicated by a variety of severe, organ-specific manifestations. These include gastrointestinal hemorrhage, typhoid intestinal perforation (TIP), meningitis, myocarditis, hepatitis, and cholecystitis (5). Splenic involvement is a hallmark of enteric fever, which mostly presents as splenomegaly. Severe splenic complications, such as splenic abscess, infarction, and venous thrombosis, are rare but have been increasingly reported (6).

Spontaneous splenic rupture (SSR) accounts for <1% of all splenic ruptures; infectious causes represent ~30% of non-traumatic ruptures. SSR in the setting of enteric fever is exceptionally rare, with only a handful of cases documented in the medical literature over the past several decades. Most of these reports are associated with *S.* Typhi. To our knowledge, SSR secondary to *S.* Paratyphi A infection is even more rare. This scarcity of evidence leaves clinicians with little guidance for the diagnosis and management of this catastrophic event.

In the present report, we describe a 15-year-old male with confirmed *S.* Paratyphi A infection who developed SSR. He was successfully treated with a nonoperative management (NOM) strategy. We also review the English-language literature concerning previously reported cases of SSR in patients with enteric fever. We aim to increase clinical awareness and provide diagnostic and therapeutic suggestions for this rare complication of enteric fever.

## Case presentation

A 15-year-old male was transferred to the Department of Infectious Diseases at the Second Affiliated Hospital of Zunyi Medical University from a local hospital. He presented with a chief complaint of persistent high fever for one week and acute, severe left upper quadrant abdominal pain for 2 days. The pain lasted approximately 3 hours, was accompanied by profuse sweating and was partially alleviated by lying still. He reported no vomiting, diarrhea, constipation, or jaundice, and he denied any history of trauma. The patient resided in a country in Guizhou Province, China, with no recent travel or consumption of contaminated food. The patient also had no history of splenic disease, immunosuppression and hematologic disorders.

On admission, the patient appeared acutely ill, febrile, and in moderate distress because of abdominal pain. His vital signs included a body temperature of 39.6 °C, a blood pressure of 110/57 mmHg, a heart rate of 102 beats per minute, and a respiratory rate of 20 breaths per minute. The presence of a heart rate of 102 bpm in the setting of a 39.6 °C fever was noted as relative bradycardia, a classic sign of enteric fever.

The abdomen was soft but distended, with significant tenderness to deep palpation in the left upper quadrant, but without significant rigidity or rebound tenderness. There were no palpable masses, and the spleen was not palpable because of guarding. Bowel sounds were hypoactive. Kehr’s sign (left shoulder tip pain on palpation) was negative. There was no demonstrable shifting dullness. The rest of the exam was unremarkable.

Initial laboratory investigations were performed upon admission. A comprehensive blood panel revealed a reduced red blood cell count of 4.17 × 10^12^/L (reference range: 4.5–5.9 × 10^12^/L), although the hemoglobin concentration was within the low-normal range at 129.0 g/L. The white blood cell count was 10.13 × 10^9^/L (reference range: 4.4–11.0 × 10^9^/L), with a neutrophilic predominance. Eosinophil levels were low—another sign that fit typhoid/paratyphoid.

C-reactive protein (CRP) concentration was 109.9 mg/L (reference <8.2 mg/L). A markedly elevated D-dimer concentration of 14.38 μg/L (reference <0.5 μg/L) was detected. Liver function tests revealed mild transaminitis, with an aspartate aminotransferase concentration of 44 U/L (reference 15–40 U/L). Serum albumin concentration was low at 27.8 g/L (reference 45–55 g/L). Serological tests for common viral causes of fever and splenomegaly, including IgM antibodies against Epstein–Barr virus, human immunodeficiency virus, cytomegalovirus, rubella virus, and herpes simplex virus, were negative.

Crucially, two sets of blood cultures drawn at a local hospital prior to transfer were reported by *S.* Paratyphi A. Antimicrobial susceptibility testing performed via the Kirby–Bauer disk diffusion method confirmed that the isolate was sensitive to ceftriaxone, ciprofloxacin, and imipenem.

Abdominal ultrasound revealed splenomegaly with heterogeneous parenchyma and a subcapsular anechoic area, suggesting a hematoma. Contrast-enhanced abdominal computed tomography (CT) confirmed splenic rupture with a subcapsular hematoma, plus multiple enlarged retroperitoneal and intra-abdominal lymph nodes, peritoneal and pelvic effusion, peritonitis, bilateral small pleural effusions, and partial atelectasis of the left lower lobe. Importantly, no active contrast extravasation was detected during the arterial or portal venous phases, indicating that the bleeding was not ongoing at the time of the scan (Figure 1).

**Figure 1:**
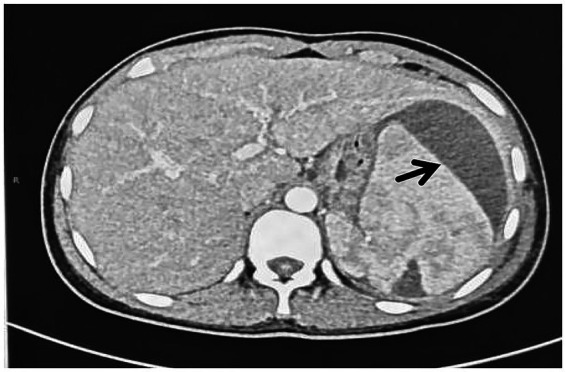
Contrast-enhanced abdominal computed tomography image showing splenic rupture with subcapsular hematoma. The arrow indicates the rupture site.

A definitive diagnosis of paratyphoid fever A complicated by SSR was established. A consultation was held with the general surgery and pediatric intensive care teams. Although peritonitis was suspected, given the patient’s hemodynamic stability following initial intravenous fluid resuscitation and the absence of active extravasation on CT, NOM was adopted.

The NOM protocol consisted of strict bed rest, intensive monitoring (including vital signs: every 30–60 min for first 24 h, then hourly for next 24 h, then every 4 h; hemoglobin: every 4 h for first 48 h, then once daily if stable; clinical exams: every 2 h for first 24 h, then once daily if stable), antibiotic therapy with intravenous imipenem (1.0 g every 8 h) and fluid and blood product support. On day 21, the patient’s hemoglobin concentration decreased to 121 g/L without concurrent hemodynamic instability. After intensive monitoring and transfusion of 2 units of packed red blood cells, his hemoglobin concentration stabilized.

The patient remained hemodynamically stable. Fever resolved after 3 days on antibiotics, and abdominal pain slowly improved. After 2 weeks of imipenem, the patient was switched to cefotaxime sodium (2.0 g every 8 h) for one week. His clinical condition continued to improve steadily over a total hospitalization of 25 days. Prior to discharge, follow-up abdominal magnetic resonance imaging (MRI) was performed, which revealed a significant reduction in the size of the subcapsular hematoma and resolution of lymphadenopathy (Figure 2A). At the three-month follow-up, the patient remained asymptomatic, and MRI showed complete resolution of the subcapsular hematoma (Figure 2B).

**Figure 2:**
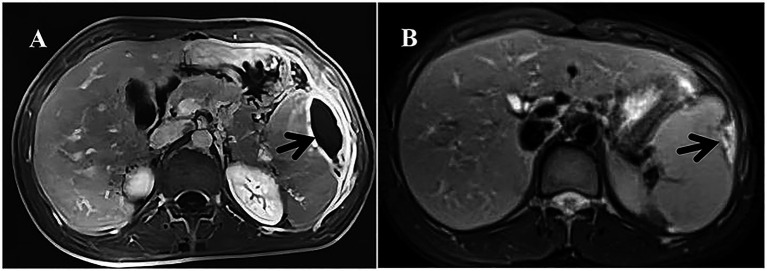
Follow-up magnetic resonance imaging (MRI) after conservative treatment, demonstrating complete resolution of the splenic hemorrhage and hematoma, with restoration of the normal splenic architecture. **(A)** MRI at 4 weeks after splenic rupture. The arrow indicates the resolution of the splenic hemorrhage and hematoma. **(B)** MR image at 12 weeks after splenic rupture. The arrow indicates the complete resolution of the splenic hemorrhage and hematoma.

## Discussion and conclusions

Paratyphoid fever has historically been considered a milder disease than typhoid fever. However, recent evidence suggests that *S.* Paratyphi A can cause equally severe clinical manifestations (5). Severe complications such as TIP and gastrointestinal hemorrhage contribute significantly to the mortality of enteric fever (7). SSR, while a known albeit rare complication of typhoid fever, remains largely undocumented in association with *S.* Paratyphi A. This report details a rare case of SSR secondary to *S.* Paratyphi A infection in an adolescent, underscoring the need for clinical vigilance.

A review of the English-language literature revealed only four reported cases of SSR in patients with typhoid fever and two cases in patients with paratyphoid fever (one due to *S.* Paratyphi A and one due to *S.* Paratyphi B infection). Among these six patients, two (33.3%) died, highlighting the potentially fatal nature of this complication. (Table 1) (7).

**Table 1:** Reported cases of spontaneous spleen rupture due to typhoid and paratyphoid fever.

Author, year	Gentle	Age	Pathogen	Management	Outcome
Ali et al., 1994 (11)	Woman	18	*S.* Typhi	NOM	Dead
Ali et al., 1994 (11)	Man	45	*S.* Typhi	Splenectomy	Survived
Ali et al., 1994 (11)	Man	52	*S.* Typhi	Splenectomy	Survived
Julià et al., 2000 (10)	Man	24	*S.* Typhi	Splenectomy	Survived
Wolthuis et al., 2020 (18)	Man	38	*S.* Paratyphi B var. Java	NOM	Survived
Wickramasinghe et al., 2012 (12)	Man	76	*S.* Paratyphi A	Splenectomy	Dead

NOM, non-operative management; *S.* Typhi, *Salmonella enterica serovar* Typhi; *S.* Paratyphi, *Salmonella enterica serovar* Paratyphi.

SSR has been reported in numerous infectious, neoplastic, and inflammatory diseases that cause sudden enlargement of the spleen. Rapidly enlarging spleens are less able to adaptively expand the capsule than chronically enlarged ones, making them more prone to rupture. Acute infectious diseases are the most common cause of SSR (8). The pathophysiology of SSR in infectious diseases is poorly understood but likely involves a multifactorial mechanism (9): (1) marked proliferation and congestion of splenic macrophages, leading to acute splenomegaly and increased capsular tension; (2) bacterial emboli causing focal inflammation, necrosis, and microabscess formation that weaken the splenic parenchyma. In addition, host-associated factors, such as transient intra-abdominal pressure spikes (e.g., coughing, vomiting, straining), can trigger rupture of a spleen under high capsular tension. Immunocompromised patients are at higher risk for invasive nontyphoidal salmonellosis, but the association between this condition and splenic rupture remains unreported.

Both *S.* Typhi infection and *S.* Paratyphi infection trigger hyperactivation of the reticuloendothelial system, resulting in acute splenomegaly, splenic abscess (8), splenic thrombosis (9), infarction (6) and SSR. In previously reported cases of SSR secondary to enteric fever, rupture occurred within 1–3 weeks of disease onset during periods of high fever (10–12), suggesting that severe systemic inflammation is one of the primary drivers of SSR in these patients (13).

Delayed diagnosis of enteric fever and its complications may significantly worsen its outcomes (14). However, early diagnosis of enteric fever and its complications is a clinical challenge, especially in nonepidemic countries. Given that the incidence of enteric fever has dramatically declined during recent decades in many countries, the rarity of the disease can lead to decreased clinical suspicion among physicians, potentially resulting in delayed diagnosis and management of its complications (15). Although two patients were reported from nonepidemic countries, both had recent travel histories from highly endemic countries (10, 12). In Guizhou Province, where our patient resided, the average annual incidence of enteric fever was 1.22 per 100,000 population, with an average annual decline of −6.50% between 2015 and 2024 (16). To our knowledge, this is the first case originating from a low-endemic country in a patient without recent travel history. The route of transmission of *S.* Paratyphi A in this patient remains unknown; it may have occurred either through unrecognized local sources (e.g., asymptomatic carriers or contaminated water sources) or through consumption of imported food from endemic regions. Our case report suggests that clinical vigilance for *S.* Paratyphi A infection must be maintained even in low-prevalence settings.

Patients with early SSR have no specific clinical manifestations and may be hemodynamically stable (Table 2). Although abdominal pain, especially acute left upper quadrant pain, may suggest SSR, it may be mistakenly attributed to other acute surgical conditions. Kehr’s sign is a characteristic clinical finding and may also be absent in SSR. CT is the imaging modality of choice, as it clearly delineates the rupture site, hematoma extent, and intraperitoneal bleeding. In resource-limited settings, ultrasound can still provide valuable preliminary diagnostic information. Therefore, a high index of suspicion is essential for any patient with suspected enteric fever who develops sudden, severe abdominal pain, particularly if accompanied by signs of hemodynamic compromise.

**Table 2:** Dynamics of the patient’s clinical and laboratory parameters.

Days after admission	1	3	8	15	21	24	Reference
Body temperature (°C)	38.4	39.5	36.9	36.4	36.9	36.7	36.1–37.1
WBC (×10^9^/L)	10.13	8.52	8.84	9.13	10.39	9.06	4.4–11.0
RBC (×10^12^/L)	4.17	4.05	4.48	4.24	3.86	4.03	4.5–5.9
Hb (g/L)	129	130	140	136	121	126	125–155
Platelets (×10^9^/L)	313	423	477	542	586	632	100–300
CRP (mg/L)	109.91	66.87	-	11.71	-	8.49	<8.2

CRP, C reactive protein; Hb, hemoglobin; RBC, red blood cells; WBC, white blood cell.

Although it is recommended that SSR management be individualized on the basis of the patient’s hemodynamic stability and the severity of splenic injury (17), determining the treatment strategy in clinical practice remains difficult. Splenectomy is the gold standard for unstable patients; however, it is associated with a lifelong risk of overwhelming post-splenectomy infection, particularly in pediatric populations. NOM of SSR has been increasingly adopted (17), but it is associated with a risk of treatment failure. Among the six previously reported patients, four underwent splenectomy (three survived), and two received NOM (one survived), suggesting that splenectomy may be safer for the management of SSR. The success in our patient suggests that the treatment strategy should be based on hemodynamic stability, the severity of splenic injury, and, most importantly, whether the patient has ongoing bleeding.

In conclusion, SSR should be suspected in any patient with enteric fever who develops acute left upper quadrant or shoulder pain, even in low-endemic countries. Early contrast-enhanced CT is essential for prompt diagnosis and treatment planning. A multidisciplinary approach and individualized management based on hemodynamic status and bleeding activity are key to optimizing outcomes.

## Data Availability

The raw data supporting the conclusions of this article will be made available by the authors, without undue reservation.

## References

[1] Liu G Zhang X Cao Q Chen T Hu B Shi H. The global burden of typhoid and paratyphoid fever from 1990 to 2021 and the impact on prevention and control. BMC Infect Dis. (2025) 25:919. doi: 10.1186/s12879-025-11223-8, 40665219 PMC12261836

[2] Piovani D Figlioli G Nikolopoulos GK Bonovas S. The global burden of enteric fever, 2017-2021: a systematic analysis from the global burden of disease study 2021. EClinicalMedicine. (2024) 77:102883. doi: 10.1016/j.eclinm.2024.102883, 39469533 PMC11513656

[3] Mogasale VV John J Sahai N Ray A Farooqui HH Mogasale V . Burden of typhoid fever and antimicrobial resistance in India (2023): a modelling study. Lancet Reg Health Southeast Asia. (2026) 44:100714. doi: 10.1016/j.lansea.2025.100714, 41551375 PMC12808886

[4] Huang S Yan M Kan B. Prevalence and influencing factor analysis of typhoid/paratyphoid fever - China, 2011-2020. China CDC Wkly. (2024) 6:493–8. doi: 10.46234/ccdcw2024.095, 38854465 PMC11154109

[5] Manesh A Meltzer E Jin C Britto C Deodhar D Radha S . Typhoid and paratyphoid fever: a clinical seminar. J Travel Med. (2021) 28:taab012. doi: 10.1093/jtm/taab012, 33550411

[6] Kumar V Lamba AS Jora B Gupta M Kaur H. Splenic infarction: an unusual complication of salmonella infection. Cureus. (2025) 17:e94171. doi: 10.7759/cureus.94171, 41209881 PMC12595516

[7] Marchello CS Birkhold M Crump JA. Complications and mortality of typhoid fever: a global systematic review and meta-analysis. J Infect. (2020) 81:902–10. doi: 10.1016/j.jinf.2020.10.030, 33144193 PMC7754788

[8] Ganesan V Rajendran T Ponnusamy SS. Splenic abscess complicating *Salmonella paratyphi* A infection: a case report and systematic review of literature (2001-2024). J Assoc Physicians India. (2025) 73:66–72. doi: 10.59556/japi.73.0998, 40955934

[9] Saini R Jesrani G Gupta M Gupta S Chhabra A. *Salmonella paratyphi*-induced splenic vein thrombosis: a case report on infrequent cause of acute abdomen. Turk J Emerg Med. (2021) 21:210–3. doi: 10.4103/2452-2473.329625, 34849434 PMC8593428

[10] Julià J Canet JJ Lacasa XM González G Garau J. Spontaneous spleen rupture during typhoid fever. Int J Infect Dis. (2000) 4:108–9. doi: 10.1016/s1201-9712(00)90104-8, 10737849

[11] Ali G Kamili MA Rashid S Mansoor A Lone BA Allaqaband GQ. Spontaneous splenic rupture in typhoid fever. Postgrad Med J. (1994) 70:513–4. doi: 10.1136/pgmj.70.825.513, 7937433 PMC2397680

[12] Wickramasinghe DP Samarasekara DN. Splenic infarction and spontaneous rupture in a patient with paratyphoid. Ceylon Med J. (2012) 57:130–1. doi: 10.4038/cmj.v57i3.4709, 23086034

[13] Veronese P Pappalardo M Maffini V Rubini M Giacometti A Ruozi MB . Severe typhoid fever complicated by superior mesenteric and splenic vein thrombosis. Infect Dis Rep. (2023) 15:377–85. doi: 10.3390/idr15040038, 37489392 PMC10366850

[14] Antillón M Warren JL Crawford FW Weinberger DM Kürüm E Pak GD . The burden of typhoid fever in low- and middle-income countries: a meta-regression approach. PLoS Negl Trop Dis. (2017) 11:e0005376. doi: 10.1371/journal.pntd.0005376, 28241011 PMC5344533

[15] Cruz Espinoza LM McCreedy E Holm M Im J Mogeni OD Parajulee P . Occurrence of typhoid fever complications and their relation to duration of illness preceding hospitalization: a systematic literature review and meta-analysis. Clin Infect Dis. (2019) 69:S435–s448. doi: 10.1093/cid/ciz477, 31665781 PMC6821330

[16] Xiang XY Yang JY Huang H Yu C Li SJ Yao GH. Analysis on epidemiological characteristics of typhoid and paratyphoid fever and patient medical treatment seeking flow in Guizhou province, 2015-2024. Zhonghua Liu Xing Bing Xue Za Zhi. (2015) 47:649–58. doi: 10.3760/cma.j.cn112338-20250901-00617, 42020166

[17] Fransvea P Costa G Massa G Frezza B Mercantini P BaIducci G. Non-operative management of blunt splenic injury: is it really so extensively feasible? A critical appraisal of a single-center experience. Pan Afr Med J. (2019) 32:52. doi: 10.11604/pamj.2019.32.52.15022, 31143357 PMC6522183

[18] Wolthuis DF Bosboom RW Hassing RJ. Spontaneous splenic rupture in an ill returned traveller. Eur J Case Rep Intern Med. (2020) 7:001891. doi: 10.12890/2020_001891, 33312999 PMC7727630

